# Estimating indirect mortality impacts of armed conflict in civilian populations: panel regression analyses of 193 countries, 1990–2017

**DOI:** 10.1186/s12916-020-01708-5

**Published:** 2020-09-10

**Authors:** Mohammed Jawad, Thomas Hone, Eszter P. Vamos, Paul Roderick, Richard Sullivan, Christopher Millett

**Affiliations:** 1 Public Health Policy Evaluation Unit, Imperial College London, 3rd Floor, Reynold’s Building, St Dunstan’s Road, Hammersmith, London, W6 8RP UK; 2 Primary Care and Population Sciences, University of Southampton, Southampton, SO16 6YD UK; 3 Institute of Cancer Policy, King’s College London & Guy’s & St Thomas’ NHS Trust, London, SE1 9RT UK

**Keywords:** Conflict, War, Health, Mortality

## Abstract

**Background:**

Armed conflict can indirectly affect population health through detrimental impacts on political and social institutions and destruction of infrastructure. This study aimed to quantify indirect mortality impacts of armed conflict in civilian populations globally and explore differential effects by armed conflict characteristics and population groups.

**Methods:**

We included 193 countries between 1990 and 2017 and constructed fixed effects panel regression models using data from the Uppsala Conflict Data Program and Global Burden of Disease study. Mortality rates were corrected to exclude battle-related deaths. We assessed separately four different armed conflict variables (capturing binary, continuous, categorical, and quintile exposures) and ran models by cause-specific mortality stratified by age groups and sex. Post-estimation analyses calculated the number of civilian deaths.

**Results:**

We identified 1118 unique armed conflicts. Armed conflict was associated with increases in civilian mortality—driven by conflicts categorised as wars. Wars were associated with an increase in age-standardised all-cause mortality of 81.5 per 100,000 population (*β* 81.5, 95% CI 14.3–148.8) in adjusted models contributing 29.4 million civilian deaths (95% CI 22.1–36.6) globally over the study period. Mortality rates from communicable, maternal, neonatal, and nutritional diseases (*β* 51.3, 95% CI 2.6–99.9); non-communicable diseases (*β* 22.7, 95% CI 0.2–45.2); and injuries (*β* 7.6, 95% CI 3.4–11.7) associated with war increased, contributing 21.0 million (95% CI 16.3–25.6), 6.0 million (95% CI 4.1–8.0), and 2.4 million deaths (95% CI 1.7–3.1) respectively. War-associated increases in all-cause and cause-specific mortality were found across all age groups and both genders, but children aged 0–5 years had the largest relative increases in mortality.

**Conclusions:**

Armed conflict, particularly war, is associated with a substantial indirect mortality impact among civilians globally with children most severely burdened.

## Background

The number of armed conflicts globally is at a record high with 182 wars and minor conflicts recorded in 2017 according to the Uppsala Conflict Data Program (UCDP) [1]. Armed conflict has been highlighted by the United Nations as a major barrier to the implementation and attainment of the Sustainable Development Goals, including improvements in global health [2]. Beyond direct and immediate casualties, armed conflict can produce enduring political instability, destroy welfare systems including health systems, and increase homelessness, unemployment, and poverty which have widespread implications for population health [3]. The destruction of key infrastructure during armed conflict as well as general toxification of the environment can adversely impact clean water and food supplies, further elevating communicable disease risk [4].

Previous cross-national research on conflict and health has methodological limitations including omitting conflicts that occur between non-state groups [5–16], conceptualising the wide spectrum of conflict types and intensities as a simple binary explanatory variable [11–15, 17–20], and assessing only immediate or short-term impacts [5–16, 18–23]. Statistical models often lack grounding in a theoretical framework and therefore omit important confounding variables that may explain the association between armed conflict and health [6, 14, 16–18, 22]. Models are also prone to a bias in cases where the number of battle-related deaths determines both the armed conflict explanatory variable and health outcome [6–13, 16, 24, 25]. Studies frequently restrict their analyses to an examination of communicable diseases [5, 6, 15, 17, 18, 22] and sub-Saharan Africa [5, 14, 17, 19, 21–25] which limits generalisability to other conditions and settings.

A comprehensive global assessment of armed conflict and health using robust approaches and addressing these limitations is therefore past due. This study aims to assess whether armed conflict of different types and intensities indirectly affects civilian mortality in the short and long term, and to determine inequalities in effect across population sub-groups. We improve on the literature by using a long time period of analysis (1990 to 2017), assessing all conflicts globally irrespective of state involvement, conceptualising conflicts using four different explanatory variables, reconstructing mortality estimates to avoid using battle-related deaths in both the exposure and outcome, and assessing effects by age- and sex-stratified causes of death up to 10 years after conflict.

## Methods

### Design and setting

This study used longitudinal (panel) regression models to assess the impact of armed conflict on mortality rates in 193 countries between 1990 and 2017. Country was the unit of analysis. Longitudinal regression methods are frequently employed in health and economic studies to examine dynamic and transitional associations over time using routine data; examples include the association between democratisation and recession on health [26, 27], and between government health spending on the incidence of disease [28]. Fixed effects specifications were employed, which adjust for time-invariant country-level factors, and can be considered similar to differences-in-differences approaches with multiple time points.

### Data

We combined several datasets for our analysis. We obtained armed conflict data from the UCDP Georeferenced Event Dataset (GED) Global v19.1 [29]. The UCDP has collected country-year armed conflict data since 1946, and event-specific data since 1989, which have been used extensively for research purposes [5, 10, 12, 17, 18, 21]. The UCDP GED provides the best estimate of the number of battle-related deaths at the village-day level for all armed conflicts that meet the UCDP definition of having at least 25 battle-related deaths per calendar year [29]. We took data from 1990 to 2017 and converted it into a country-year dataset. The UCDP GED assigns armed conflict events in Palestine to Israel, misclassifying the former as being conflict-free, so both countries were removed from the dataset. Further details on the UCDP methodology are shown in Additional file 1 (Paragraph 1.1).

We obtained country-level mortality data between 1990 and 2017 from the Global Burden of Disease (GBD) study [30]. The GBD study utilises data from various sources to produce internally consistent, statistically modelled annual estimates of mortality by causes of death for each country. Estimates are not restricted to country citizens or nationals, but include migrants and refugees, including those displaced by conflict. The GBD excludes direct battle-related deaths from initial all-cause mortality calculations, reintroducing them in later calculations. Thus, battle-related deaths are listed as a separate cause of death (Category C3.3: terrorism and armed conflict) [30]. The GBD is appropriate for studying excess deaths from armed conflict because no indirect consequences of armed conflict are built into the underlying mortality estimations. Although GBD data are modelled with smoothing functions, investigation of mortality trends for selected countries experiencing conflict shows GBD smoothing functions did not obscure abrupt changes in mortality and are unlikely to introduce bias to this analysis (Additional file 1: Fig. S1).

We developed a conceptual framework (Additional file 2) using existing conflict and health literature which informed the selection of covariates. The framework consisted of seven pathways through which armed conflict may affect civilian mortality, and seven drivers of conflict which were used as potential confounders. To capture changes in country wealth and income, we used the gross domestic product (GDP) per capita in current US dollars, available from the World Bank [31], and membership of the Organisation for Economic Co-operation and Development (OECD) [32]. For changes in the degree of democratisation, we used the Varieties of Democracy (V-Dem) dataset, specifically the Multiplicative Polyarchy Index (continuous variable, range 0 to 1, created by multiplying five core components of electoral democracy) [33]. We used V-Dem rather than the more commonly used POLITY IV [5, 7, 10, 12, 13] due to better data completeness. For changes in demographic factors, we used data on the proportion of people living with a density > 1000 people/km^2^, the proportion living in urban areas (both taken from the Institute for Health Metrics and Evaluation (IHME) [34]), and the age dependency ratio (ratio of under 15s and over 64s to the working population) taken from the World Bank [31]. For changes in the average levels of educational attainment in countries, we used the mean years of educational attainment per capita, separately for males and females, available from the IHME [34]. We captured changes in ethnic group composition using the Historical Index of Ethnic Fractionalisation Dataset, which corresponds to the probability that two randomly drawn individuals within a country are not from the same ethnic group [35]. Finally, to capture changes and shocks in climate-related factors, we took data from the Emergency Disasters Database to control for the presence of droughts and earthquakes [36], and the IHME to control for the population-weighted mean temperature and the proportion of people living in the 5th quintile of annual rainfall. We were unable to find a suitable dataset for income inequality, although it is plausible that its effects are captured through GDP per capita and educational attainment. Unlike previous research, we did not adjust for health expenditure [5, 7–9], the prevalence of HIV/AIDS [11, 13], and refugee movement [5, 15] as these can be considered mediators (i.e. factors on the causal pathway between conflict and health) rather than confounders.

### Measures

Our main outcome measures were all-cause and cause-specific mortality rates, as reported by the GBD study [30], but with deaths due to terrorism and armed conflict (Category C3.3) removed. Removing battle-related deaths from all-cause mortality prevents the bias of including battle-related deaths in both the explanatory variable and outcome measure which is commonly found in previous studies [6–13, 16, 24, 25]. The GBD study categorises cause-specific mortality into first-order (communicable, maternal, neonatal, and nutritional diseases; non-communicable diseases [NCDs]; injuries) and second-order causes [30].

As explanatory variables for armed conflict, we explored four different specifications. Firstly, we used a binary variable indicating the presence of armed conflict per country-year observation (0 = no [< 25 battle-related deaths per country-year], 1 = yes [≥ 25 deaths]) as per the UCDP [1]. This approach is limited as it groups all conflicts together regardless of intensity, so a second specification was the rate of battle-related deaths per 100,000 population as a continuous measure. We used the rate rather than absolute battle-related deaths to avoid bias from emphasising small conflicts in populous countries which are unlikely to exert country-wide effects. Thirdly, as previous research has shown the relationship between battle deaths and civilian mortality to be non-linear [24], we explored quintiles of the rate of battle-related deaths per 100,000 population. Fourthly, we used intensity cutoffs as per the UCDP [1] to create a categorical conflict variable (0 = no conflict [< 25 battle-related deaths per country-conflict-year], 1 = minor conflict [25–999 deaths], 2 = war [≥ 1000 deaths]). We used county-conflict-year for this final specification to prevent misclassifying large countries with multiple small conflicts as war-affected (should the total of these conflicts be greater than 1000 battle deaths).

### Statistical analysis

We described our sample using frequencies and means and reported the rates of battle-related deaths and civilian mortality by the different conflict explanatory variables. We presented graphical time trends in the number of conflicts, the number and rate of battle-related deaths, and the mean battle-related deaths per country.

We then used fixed effects linear panel regression methods to assess the relationship between armed conflict and mortality, which was estimated using the following equation:

$$ {\mathrm{Mortality}}_{it}={\beta}_0+{\beta}_1{\mathrm{Conflict}}_{i\left(t-1\right)}+{\beta}_2{\mathrm{Covariates}}_{it}+i+t+{u}_{it} $$

where *i* is the country, *t* is the year, and *u* is the error term representing unexplained variation. We chose fixed effects over random effects given the likelihood that our error term *u* was correlated with our covariates (a key assumption that would be violated in random effects) and as indicated by the Hausman test. Fixed effects specifications control for time-invariant observed and unobserved country-level factors (as denoted by *i* in the equation above) and therefore only assess within-country associations rather than between-country associations. Model fit was assessed using scatter plots and post-regression diagnostics (including the variance inflation factor (VIF), studentised residuals, stem and leaf plots, Cook’s D, and DFITS) to identify collinear variables and outliers. Due to collinearity with female education (VIF > 30) and superior goodness-of-fit, we only included male education. Four data points were considered outliers and dropped (from the model only using a continuous measure for armed conflict) based on having large residuals and high leverage: Rwanda 1994, Bosnia and Herzegovina 1995, Congo 1997, and Eritrea 1999. Four of twelve covariates had missing data: age dependency ratio (4.3%), GDP per capita (5.8%), Multiplicative Polyarchy Index (11.7%), and Ethnic Fractionalisation Index (31.6%); the latter two were omitted from main analyses due to high levels of missing data, but their effects tested in sensitivity analyses (see below).

Our first models separately tested the association between the four explanatory variable specifications for armed conflict and age-standardised all-cause mortality, adjusting for ten covariates (GDP per capita, OECD member, population density, urban living, age dependency ratio, male education, temperature, rainfall, earthquakes, droughts), and year-country fixed effects. Mortality rates were standardised using the GBD world population standard. We included country-clustered Huber-White robust standard errors to account for possible heteroscedasticity and serial correlation. In all models, we lagged the armed conflict variable by 1 year unless otherwise stated to capture indirect deaths in the year after.

Because higher intensity armed conflicts drove the associations, our second models employed the same specification as above but using only the categorical armed conflict exposure variable for war (we present “war” vs. no conflict). These models used age-standardised all-cause and cause-specific mortality rates (first- and second-order causes; the latter helping to explain underlying drivers) and age- and sex-stratified all-cause and cause-specific mortality rates (first-order causes only). We then assessed the lagged effects of war (from 2 to 10 years with a separate model for each year lag) on age-standardised all-cause and cause-specific (first-order causes only) mortality to account for the varying progression of disease pathologies. We used the models’ “war” beta coefficients to calculate the absolute and relative change in civilian mortality and used post-estimation commands to calculate the number of civilian deaths.

In additional analyses, we explored whether armed conflicts that involved particular actors (“armed conflict actor type”) had differential associations with civilian mortality. In accordance with UCDP definitions, we categorised each armed conflict as being state-based (at least one actor is the state of a country), non-state (no actors are the state of a country), or one-sided violence (one actor in armed conflict with civilians). We analysed all four explanatory variable specifications for conflict (i.e. binary, continuous, quintiles, and categorical) by armed conflict actor type, and for countries with only one actor type in each year, we interacted actor type and the rate of battle-related deaths, leaving all specifications identical to prior models.

### Sensitivity analyses

We undertook multiple sensitivity analyses to check the robustness of findings. First, we tested alternative model specifications with sequential addition of covariates, addition of new covariates that contained high rates of missing data, and random effects. Second, to address the possibility of misclassification bias between deaths due to armed conflict and deaths due to homicide, we repeated our main model removing deaths from interpersonal violence. Third, we used an alternative measure of armed conflict exposure derived from the Major Episodes of Political Violence dataset compiled by the Centre for Systemic Peace [37]. This dataset captures major conflicts globally, and its binary conflict variable (0 = no conflict, 1 = conflict) is 97.6% specific and 97.7% sensitive to the UCDP “war” binary variable (0 = no conflict, 1 = war). More details about how this dataset compares to the UCDP are presented in Additional file 1: Paragraph 1.2. All analyses were conducted in Stata 15.

## Results

### Sample description

**Fig. 1:**
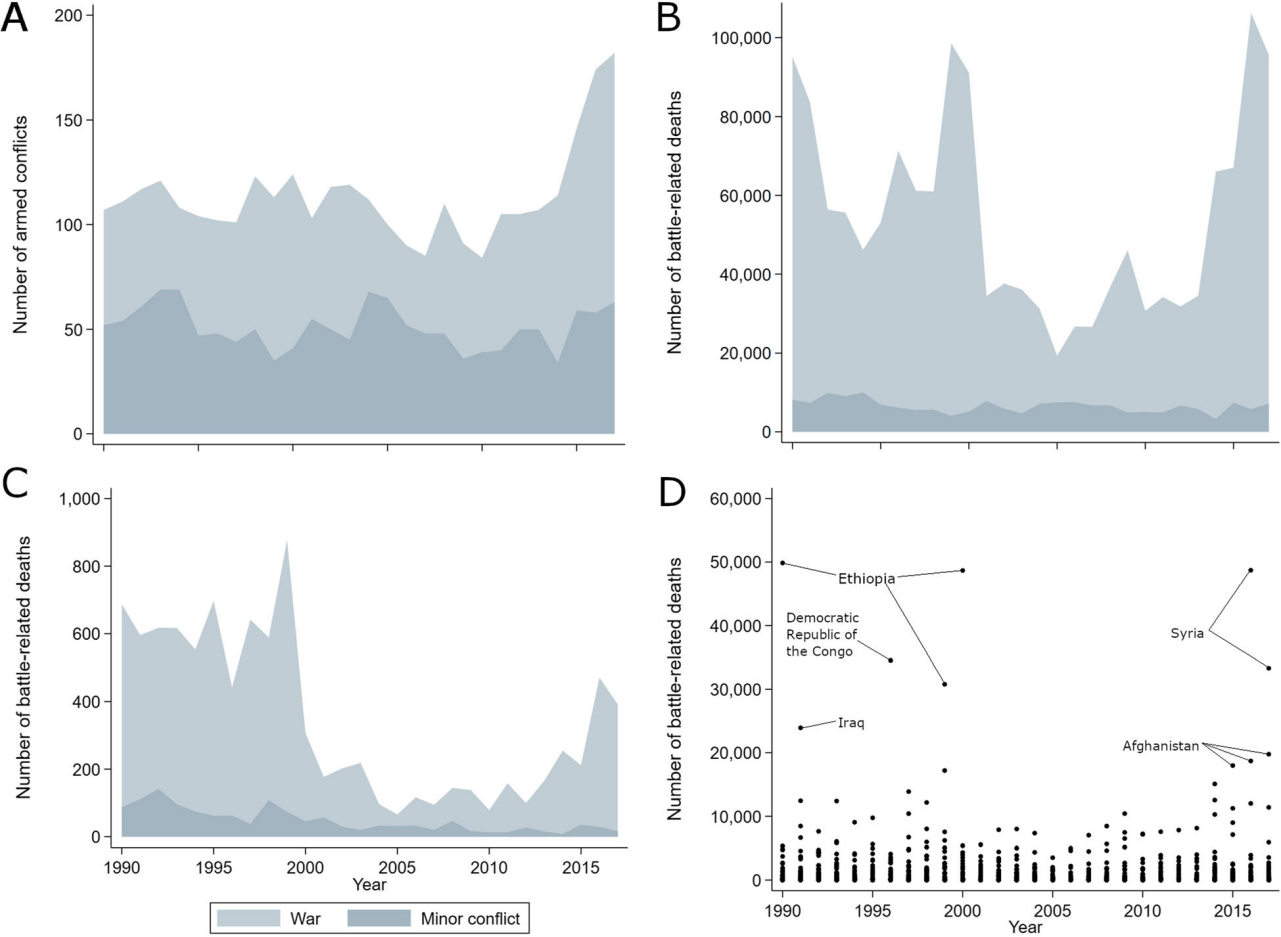
Trends in armed conflict Figures 1B-1D do not include 500,907 deaths from the 1994 Rwanda genocide

Between 1990 and 2017, the UCDP recorded 1118 unique armed conflicts involving 102 countries. Conflicts accounted for 1020 of 5404 (18.9%) country-year observations in the dataset, of which 686 (67.3%) were minor conflicts and 334 (32.7%) were wars (Table 1). The highest number of conflicts in any year was 182, recorded in 2017, of which 60.3% were categorised as war (Fig. 1a). Despite an increase in the number (Fig. 1b) and rate (Fig. 1c) of battle-related deaths annually since 2014, these figures were lower than during the decade from 1990 to 2000. Countries in conflict had an average of 19.2 battle-related deaths per 100,000 population, and this was substantially higher for countries in war (54.7) than in minor conflict (2.0) (Table 1). Countries in conflict had an average mortality rate of 1273 per 100,000 population compared with 962 in those without conflict; this was only slightly higher for countries in war (1355) than in minor conflict (1233). By country quintiles of the rate of battle-related deaths, the average mortality rate increased from 998 in the first quintile to 1502 in the fifth (Table 1).

**Table 1:** Description of data used in the study

	**Countries (*N*)**	**Observations (*N*)**	**Battle-related deaths per 100,000 population (mean, SD)**	**Civilian mortality rate per 100,000 population (mean, SD)**
**Total**	193	5404	3.63 (135.52)	1020.84 (484.54)
**Armed conflict exposure**^**1**^				
No	186	4384	0.01 (0.08)	962.11 (459.71)
Yes	102	1020	19.23 (311.58)	1273.26 (507.18)
- Minor conflict	99	686	1.96 (4.09)	1233.39 (492.27)
-War	48	334	54.72 (543.30)	1355.15 (527.87)
**Quintile of exposure**^**2**^				
- None	177	3986	–	943.81 (449.20)
- First	65	284	0.02 (0.01)	998.09 (435.49)
- Second	80	284	0.13 (0.05)	1179.62 (473.60)
- Third	68	283	0.51 (0.18)	1197.73 (463.11)
- Fourth	71	285	2.44 (1.18)	1310.55 (501.44)
- Fifth	50	283	66.35 (589.68)	1501.68 (553.86)
**Covariates**	**Countries (*N*)**	**Observations (*N*)**	**Mean**	**SD**
GDP per capita	190	5082	9825.06	15,484.47
OECD membership	193	5404	0.15	0.36
Population density	193	5404	0.29	0.21
Urbanisation	193	5404	0.56	0.19
Age dependency ratio	185	5171	65.39	19.61
Male education	193	5404	8.21	3.29
Temperature	193	5404	14.12	13.32
Rainfall	193	5404	0.45	0.42
Earthquake	193	5404	0.09	0.28
Drought	193	5404	0.08	0.27
Multiplicative Polyarchy Index	172	4774	0.52	0.27
Ethnic Fractionalisation Index	156	3703	0.45	0.26

GDP per capita is in current US dollars. Population density represents the percentage of the population living in a density of > 1000 people/km^2^. Urbanisation represents the percentage of the population living in urban areas. The age dependency ratio represents the percentage of the population younger than 15 years and older than 64 years per 100 working-age population. Male education is expressed as years per capita and is age-standardised. Temperature is in degrees Celsius and is the mean population-weighted annual temperature. Rainfall represents the percentage of the population living in the top world quintile of annual rainfall. Earthquake and drought are binary variables representing their absence or presence

^1^Based on the number of battle-related deaths per country-conflict-year: no, < 25; yes, ≥ 25; minor conflict, 25–999; war, ≥ 1000. ^2^Based on the number of battle-related deaths per 100,000 population per country-year

### All-cause mortality

**Fig. 2:**
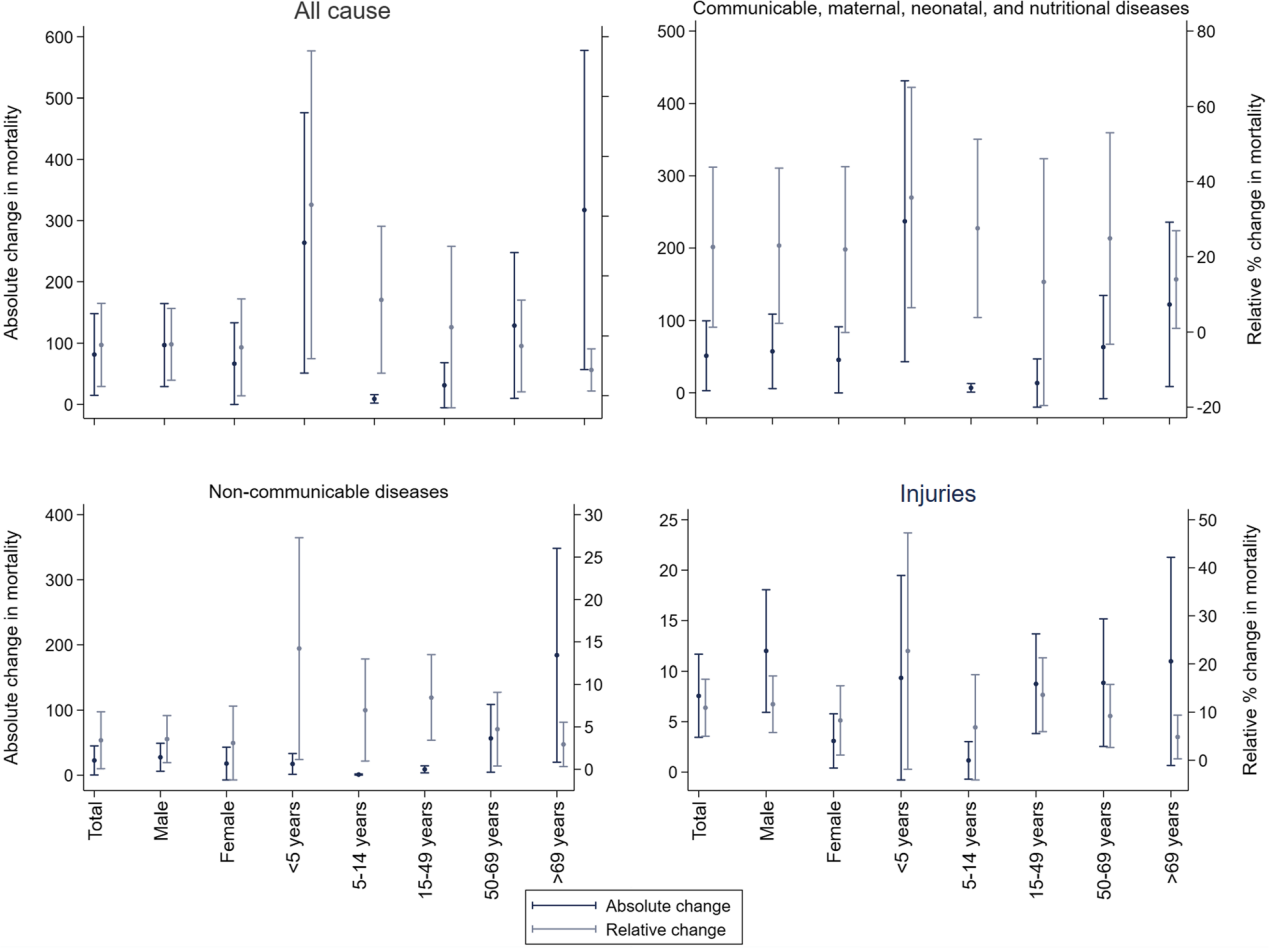
The association between war and mortality

Armed conflict was positively associated with an increase in the age-standardised all-cause mortality rate among all four explanatory variable specifications (Table 2). Each battle-related death per 100,000 population was associated with an increase in civilian mortality by 1.8 per 100,000 population (*β* 1.8, 95% CI 0.6–3.0). The fifth quintile of armed conflict exposure (i.e. > 5 battle-related deaths per 100,000 population) was associated with an increase in civilian mortality of 99.2 per 100,000 population (*β* 99.2, 95% CI 28.2–170.3), with other quintiles showing no significant association. Only war was associated with an increase in civilian mortality of 81.5 per 100,000 population (*β* 81.5, 95% CI 14.3–148.8) (Fig. 2a) with no significant association for minor conflict. The relationship was consistent when armed conflict was expressed as a binary variable (*β* 26.4, 95% CI − 12.9–65.8) although was non-significant. Post-estimation analysis predicted 29.4 million (95% CI 22.1–36.6) civilian deaths were attributable to all wars over the study period.

**Table 2:** The association between armed conflict and age-standardised all-cause mortality, 1990–2017

	Binary	Continuous (battle-related deaths per 100,000)	Quintiles of conflict	Categories of conflict
Armed conflict variable	*β* (95% CI)	*β* (95% CI)	*β* (95% CI)	*β* (95% CI)
No (< 25 battle deaths/year)	0.00			
Yes (≥ 25 battle deaths/year)	26.44 (− 12.88, 65.76)			
Battle deaths/100,000 population		1.76 (0.56, 2.96)**		
None (0 battle deaths/year)			0.00	
First quintile			− 11.59 (− 31.62, 8.44)	
Second quintile			− 16.24 (− 42.50, 10.02)	
Third quintile			8.81 (− 34.20, 51.82)	
Fourth quintile			27.26 (− 23.31, 77.83)	
Fifth quintile			99.21 (28.16, 170.26)**	
None (< 25 battle deaths/year)				0.00
Minor conflict (25–999 battle deaths/year)				13.05 (− 22.74, 48.84)
War (≥ 1000 battle deaths/year)				81.52 (14.30, 148.75)*
**Covariates**				
GDP per capita	0.00 (− 0.00, 0.00)	0.00 (− 0.00, 0.00)	0.00 (− 0.00,0.00)	0.00 (− 0.00, 0.00)
OECD membership	− 38.32 (− 96.14, 19.50)	− 38.23 (− 95.85, 19.40)	− 40.38 (− 97.92, 17.16)	− 39.67 (− 97.20, 17.86)
Population density	− 20.00 (− 345.62, 305.61)	− 24.52 (− 350.11, 301.07)	− 36.48 (− 366.3, 293.3)	− 20.36 (− 342.29, 301.58)
Urbanisation	− 16.58* (− 30.93, − 2.22)	− 16.45* (−30.70, − 2.21)	−15.75* (− 29.91, − 1.60)	− 16.17* (− 30.33, − 2.00)
Age dependency ratio	− 2.49 (− 5.52, 0.53)	− 2.36 (− 5.37, 0.65)	− 2.50 (− 5.49, 0.50)	− 2.45 (− 5.46, 0.57)
Male education	− 34.09 (− 87.46, 19.29)	− 32.63 (− 85.53, 20.27)	− 34.46 (− 86.93, 18.02)	− 33.80 (− 86.85, 19.25)
Temperature	− 7.10 (− 24.75, 10.55)	− 6.93 (− 24.54, 10.68)	− 7.31 (− 25.29, 10.68)	− 7.16 (− 24.87, 10.55)
Rainfall	19.97 (− 14.97, 54.91)	20.71 (− 13.70, 55.11)	20.38 (− 13.19, 53.95)	21.18 (− 12.67, 55.04)
Earthquake	4.79 (− 16.49, 26.06)	5.30 (− 16.12, 26.72)	6.13 (− 15.28, 27.54)	6.12 (− 15.29, 27.52)
Drought	1.38 (− 14.50, 17.26)	1.84 (− 13.17, 16.85)	1.31 (− 14.31, 16.92)	1.27 (− 14.27, 16.82)
Observations	4746	4746	4746	4746
Countries	183	183	183	183

GDP per capita is in current US dollars. Population density represents the percentage of the population living in a density of > 1000 people/km^2^. Urbanisation represents the percentage of the population living in urban areas. The age dependency ratio represents the percentage of the population younger than 15 years and older than 64 years per 100 working-age population. Male education is expressed as years per capita and is age-standardised. Temperature is in degrees Celsius and is the mean population-weighted annual temperature. Rainfall represents the percentage of the population living in the top world quintile of annual rainfall. Earthquake and drought are binary variables representing their absence or presence. All armed conflict variables were lagged by 1 year

Note: * *p* < 0.05, ** *p* < 0.01, *** *p* < 0.001. Robust standard errors were employed. Each column is the output from one panel regression with fixed effects adjusted for the covariates in the table in addition to year dummies (not shown). Coefficients are interpreted as the change in all-cause mortality per 100,000 following a change in one unit of the independent variable

### Cause-specific mortality

As associations identified were driven by the more intense armed conflicts, we used war as our explanatory variable in cause-specific analyses. Wars were associated with an increase in the age-standardised mortality rate from communicable, maternal, neonatal, and nutritional diseases by 51.3 per 100,000 population (*β* 51.3, 95% CI 2.6–99.9; relative increase 22.6%, 95% CI 1.3–43.8) (Fig. 2b and Additional file 3: Table S3). The association was driven by increased deaths from respiratory infections and tuberculosis, neglected tropical diseases and malaria, enteric infections, and maternal and neonatal disorders (Additional file 4: Fig. S4). Post-estimation analysis estimated 21.0 million (95% CI 16.3–25.6) civilian deaths from these diseases were related to all wars over the study period.

Wars were also associated with an increased civilian mortality from NCDs by 22.7 per 100,000 population (*β* 22.7, 95% CI 0.2–45.2; relative increase 3.4%, 95% CI 0.1–6.8; number of deaths 6.0 million, 95% CI 4.1–8.0) (Fig. 2c and Additional file 3: Table S3). The association was driven by increased cardiovascular diseases, diabetes and kidney diseases, neoplasms, and digestive diseases (Additional file 4: Fig. S4). Wars were associated with increased civilian mortality from injuries by 7.6 per 100,000 population (*β* 7.6, 95% CI 3.4–11.7; relative increase 10.9%, 95% CI 5.0–16.9; number of deaths 2.4 million, 95% CI 1.7–3.1) (Fig. 2d and Additional file 3: Table S3). The association was driven by self-harm and interpersonal violence (Additional file 4: Fig. S4).

### Differences by sex

Wars were associated with increases in all-cause mortality for males (*β* 97.0, 95% CI 28.7–165.2; relative increase 8.6%, 95% CI 2.6–14.6), but not females (*β* 66.6, − 0.6–133.8) (Fig. 2a and Additional file 5: Table S5.1-S5.2). This pattern remained similar for mortality from communicable, maternal, neonatal, and nutritional diseases (Fig. 2b and Additional file 5: Table S5.1-S5.2) and for NCD mortality (Fig. 2c and Additional file 5: Table S5.1-S5.2). Wars were associated with higher increases in mortality from injuries in males (*β* 12.0, 95% CI 5.9–18.1) than in females (*β* 3.1, 95% CI 0.4–5.8), but their relative increases were similar (males 11.7%, 95% CI 5.8–17.6; females 8.3%, 95% CI 1.1–15.5) (Fig. 2d and Additional file 5: Table S5.1-S5.2).

### Differences by age group

Absolute increases in all-cause mortality associated with war were largest among children under the age of 5 years (*β* 263.7, 95% CI 49.7–477.7) and adults over the age of 69 years (*β* 317.3, 95% CI 55.2–579.4) (Fig. 2a and Additional file 6: Table S6.1-S6.5). This pattern was consistent for deaths from communicable, maternal, neonatal, and nutritional diseases (Fig. 2b) and for deaths from injuries (Fig. 2d). However, for NCD mortality, the absolute effects were largest among adults over the age of 69 years only (*β* 184.1, 95% CI 18.8–349.3) (Fig. 2c and Additional file 6: Table S6.1-S6.5). Relative increases in all-cause and cause-specific mortality associated with war were consistently largest in children aged under 5 years and decreased incrementally as age increased (Fig. 2 and Additional file 6: Table S6.1-S6.5).

### Lagged effects

Following the onset of war, increases in mortality persisted for 2 years for all-cause mortality and 3 years for mortality from injuries (Additional file 7: Fig. S7). Wide confidence intervals do not preclude longer lagged effects for these outcomes nor for mortality from communicable, maternal, neonatal, and nutritional diseases and mortality from NCDs.

### Actor type

Countries experiencing one-sided violence had a higher average rate of battle-related deaths (21.1) than countries experiencing state-based (8.1) and non-state (2.0) conflicts (Additional file 8: Table S8.1.) This pattern remained similar for armed conflict actor types categorised as war or as quintiles. A visual inspection of scatter plots by armed conflict actor type showed no clear differences in the relationship between the rate of battle-related deaths and civilian mortality (Additional file 8: Fig. S8.1). In a sub-sample of 4133 country-years that only had one armed conflict actor type, no significant association between actor type and the rate of battle-related deaths was found (Additional file 8: Table S8.2) indicating that differential effects in mortality by armed conflict actor type is determined by the intensity of battle rather than the actors involved.

### Sensitivity analysis

Sensitivity analyses demonstrated the robustness of our findings. Alternative model specifications including random effects (Additional file 9: Table S9.1) did not change our results. The sequential addition of covariates demonstrated model stability (Additional file 9: Table S9.2). The addition of two new covariates with substantial missing data, the Multiplicative Polyarchy Index and the Ethnic Fractionalisation Index, reduced the number of country-year observations from 4754 to 3381 and the number of countries from 183 to 152. However, the model’s output (war coefficient: *β* 87.7, 95% CI 19.1–156.1) was otherwise similar to our main findings, showing the inclusion of additional potential confounders did not alter our findings. An analysis of age-standardised all-cause mortality omitting deaths from interpersonal violence to assess misclassification bias also showed concordant results (Additional file 9: Table S9.3; war coefficient: *β* 78.3, 95% CI 11.1–145.5). Replacing the UCDP dataset with the MEPV dataset also showed a positive association between armed conflict and all-cause mortality (*β* 51.2, 95% CI − 6.8–109.1) although this did not attain statistical significance due to the MEPV dataset including many low-intensity conflicts in its binary conflict variable (mean rate of battle-related deaths compared with the UCDP, 22.7 vs. 53.7; Additional file 1: Table S2.2.).

## Discussion

This study is the first to quantify the indirect impact of armed conflict on mortality in civilian populations globally. We found that armed conflict, irrespective of how it is measured, was positively associated with all-cause mortality. While conflicts can involve a range of different state and non-state actors, we found that it is the intensity of conflict rather than who is involved which determines civilian mortality impact. Wars, the most intense form of armed conflict, were associated with an increase in age-standardised mortality of civilians from all causes by an average of 81.5 per 100,000 population which equated to approximately 29.4 million deaths between 1990 and 2017. Communicable, maternal, neonatal, and nutritional diseases (21.0 million deaths); NCDs (6.0 million deaths); and injuries (2.4 million deaths) all contributed to increased civilian deaths associated with wars. Effect estimates were disproportionately larger for children aged under 5 years, regardless of the cause of death.

Our finding of increased war-associated deaths from respiratory, enteric, and neglected tropical diseases is explained by difficulties in maintaining sanitation, avoiding overcrowded living arrangements, and continuing coverage of immunisations following armed conflict and forced displacement [38]. We also found increased deaths from maternal and neonatal disorders, a finding supported by studies from sub-Saharan Africa [21, 24, 25], which may reflect less access to skilled birth attendants and health centres for delivery. NCDs have been highlighted as a particular health concern in modern-day protracted conflicts [39], especially in the growing list of conflict-affected countries where the epidemiological transition has occurred and the baseline burden of NCDs is already substantial. Our finding of increased NCD deaths associated with war supports the results of a recent review on the topic which linked both the destruction of health systems and changes to individual behaviours (e.g. increased tobacco and alcohol use) to increased cardiovascular disease risk [39]. Our positive associations between war and injuries, including self-harm and interpersonal violence, also align with recently published literature [40, 41].

This study uses robust statistical methods to comprehensively quantify the impact of armed conflict on excess mortality globally over the last 30 years. We provide a detailed examination of this relationship by armed conflict exposure, across causes of death, and explore differential impacts by population sub-groups. However, there are several limitations. The UCDP relies on journalism and may not include smaller armed conflicts that are not considered newsworthy; however, our findings are driven mainly by the most intense conflicts so it is unlikely that small-scale omissions would affect our results. Other armed conflict datasets such as ACLED are generally incomplete on a global scale and are less suitable for longitudinal cross-national research, e.g. ACLED commenced data collection for Africa in 1997, Asia in 2010, the Middle East in 2016, and Europe in 2018 [42].

Another limitation is data processing of mortality estimates from the GBD. The modelling methods of the GBD study are complex, and smoothing processes may mask sudden shifts in mortality. While we have shown the presence of civilian mortality spikes in conflict-affected countries that justify our methodological approach (Additional file 1: Fig. S1), we cannot exclude the possibility that data smoothing processes may produce conservative estimates. However, our findings are in line with recent estimates of indirect deaths from several armed conflicts. Applying our model coefficient to the 2003 US-led invasion of Iraq would estimate 20,902 indirect deaths in 2004, which is slightly higher than but similar to Burnham et al.’s average estimate of 16,181 per year [43]. Similarly, our model would estimate 21,603 indirect deaths in Yemen in 2016, whereas a recent UNDP report on the impact of armed conflict estimated an average of 32,750 per year [44]. While other cross-national health data are available, including from the World Health Organization and World Bank, these also have shortcomings in relation to incompleteness and reporting inconsistencies, especially in conflict-affected settings. The GBD is the only source of health data to our knowledge that provides first- and second-order causes of death that can support an examination of the broader mortality patterns observed.

Our analysis is also ecological at the country-level, and our findings may mask focal points of conflict intensity sub-nationally and the likely large inequalities in impact across populations. Fixed effects panel regression produces effect sizes interpreted in relation to the “average country” rather than a global average and is more robust than that typically used by previous cross-national studies in this field. Panel regression models can also be potentially biased from unmeasured confounders which could explain both changes in mortality and conflict status. However, we control for a range of variables that are theoretically justified and serve as key proxies for potential unmeasured confounders. Finally, we were unable to capture the effect for conflict-displaced refugees who died in conflict-free countries, and this necessitates further investigation especially as these populations are likely to be heavily affected by conflict. Limitations relating to possible under-reporting of conflict, the use of country-level data, and data on refugees all suggest our analyses offer conservative estimates of the impact of conflict.

Quantifying the wider health burden of armed conflict is vital to draw the attention of citizens and world leaders to this important global issue. This study provides robust estimates that can help galvanise communities that fight to prevent conflict and to protect civilians. While our study reports average impacts of conflict globally, humanitarian and public health responses must be tailored to the unique context of each armed conflict. Health policy implications of armed conflict typically focus on secondary or tertiary prevention, such as integrating humanitarian aid and peace building initiatives with health system planning and reconstruction [45], and strengthening accountability for violations of international humanitarian law [46]. While these are important goals, prevention and conflict deterrence is vital. The upstream drivers of armed conflict include socioeconomic inequalities, normalisation of militarism, and small arms and light weapons (SALW) proliferation. Health professionals can play an important role in the prevention of armed conflict, including through documenting its wider physical and mental health burden. Furthermore, health professionals in all countries have the moral responsibility to use their influence and status to protect communities most affected by conflict by advocating for nonviolent and diplomatic political resolution. Public health and medical curricula worldwide should include more on the prevention of armed conflict to promote further awareness, research, and capacity building. Further research should focus on quantifying the impact of armed conflict using a broader range of mortality and morbidity health indicators, examine inequalities in impacts masked by country-level data, and identify effective strategies to preserve health systems and health-promoting institutions to reduce mortality during conflict and post-conflict periods. In particular, linkage studies of civilian mortality and morbidity, including quantitative research and victimisation surveys, in conflicts to specific risk exposures e.g. SALW and explosive remnants of war [47], can drive powerful evidence-based policy-making through national interventions and multi-lateral agreements.

## Conclusion

Armed conflict, especially the most intense types, indirectly impacts civilian mortality. Our estimates suggest that almost 30 million civilian deaths were indirectly attributable to armed conflict globally between 1990 and 2017, two thirds of which were due to communicable, maternal, neonatal, and nutritional diseases. Broader and more robust measures of civilian impacts at subnational and national levels are needed to inform policy and advocacy to prevent war and protect civilians. This could include greater use of linkage studies that incorporate data from routine health and demographic sources, exposure to conflict-specific environmental risks, and quantitative epidemiological methods such as national and subnational victimisation surveys.

## Supplementary information

**Additional file 1.** Additional methodological details.

**Additional file 2.** Conceptual framework.

**Additional file 3.** First order causes of death.

**Additional file 4.** Second order causes of death.

**Additional file 5.** Differences by sex.

**Additional file 6.** Differences by age group.

**Additional file 7.** Lags.

**Additional file 8.** Differences by actor type.

**Additional file 9.** Sensitivity analysis.

## Data Availability

All data used in this study are publically available and are cited with URLs.
