# Supplementary material for: Estimating indirect mortality impacts of armed conflict in civilian populations: panel regression analyses of 193 countries, 1990–2017
Source: BMC Med. 2020 Sep 10;18:266. doi: 10.1186/s12916-020-01708-5 (PMC7487992; doi:10.1186/s12916-020-01708-5)
Supplement: Supplementary file 1 — Additional file 1. Additional methodological details. [file 12916_2020_1708_MOESM1_ESM.docx]

**ADDITIONAL FILE 1. ADDITIONAL METHODOLOGICAL DETAILS**

**1.1. Methodology of the Uppsala Data Conflict Program**

Approximately 80% of UCDP data is sourced from news reports; search strings are run through the Dow Jones Factiva aggregator to automatically retrieve articles about individuals killed or injured, and other local and specialised news sources are added to improve coverage for identified armed conflicts.^1^ Approximately 10,000-12,000 events are coded annually by researchers using this method.^1^ Another 20% of UCDP data is sourced from non-governmental and international organisations, case studies, truth commission reports, historical archives and other sources of information.^1^ Reports are then traced back to the primary source to assess its reliability; the data are at least triple checked and coded by staff with extensive case knowledge, and subject to post estimation validation methods that ensure high quality and consistency, and are explained in more detail elsewhere.^1^

**1.2. Methodology of the Major Episodes of Political Violence**

The Major Episodes of Political Violence (MEPV) dataset is managed by the Center for Systemic Peace.^2^ MEPV defines armed conflict as the “systematic and sustained use of lethal violence by organized groups that result in at least 500 directly-related deaths over the course of the episode”, with a base rate of 100 directly-related deaths per year.^2^ It is unclear which sources of information are used to inform the MEPV dataset, although armed conflict data are researched, analysed and coded by a single author.

Each episode is categorised as either international, civil, or ethnic conflict, and depending on its intensity is then arbitrarily categorised as either violence or war. Each conflict in each country-year is rated on an 11 point ratio scale that represents the “magnitude of societal-systematic impact” (Table), which is summed if more than one conflict occurs per country-year.^3^ These scores reflect multiple factors including state capabilities, interactive intensity (means and goals), area and scope of death and destruction, population displacement, and episode duration. The scores are comparable over time and between countries.

A comparison of the rate of battle-related deaths between various armed conflict variables from UCDP and MEPV is shown in the table below.

**Table S1.1.** A comparison of categorical armed conflict variables from the UCDP and MEPV datasets

|  | MEPV | |
| --- | --- | --- |
| UCDP | No conflict | Conflict |
| No conflict | 4,261 | 97 |
| Minor conflict | 277 | 409 |
| War | 8 | 324 |
|  |  |  |
| Sensitivity/Specificity (Minor conflict + war grouped together) | 72.0/97.8 | |
| Sensitivity/Specificity (Minor conflict removed) | 97.6/97.8 | |

**Table S1.2.** A comparison of battle-related deaths between the UCDP and MEPV datasets

|  | Uppsala Conflict Data Program | | | Major Episodes of Political Violence | | |
| --- | --- | --- | --- | --- | --- | --- |
|  | Countries (N) | Observations (N) | Battle-related deaths per 100,000 population (mean, range) | Countries (N) | Observations (N) | Battle-related deaths per 100,000 population (mean, range) |
| Armed conflict exposure | | | | | | |
| No | 185 | 4,358 | 0.01 (0.00 – 3.19) | 185 | 4,546 | 0.08 (0.00 – 34.27) |
| Yes | 101 | 1,018 | 18.83 (0.01 – 9,914.43) | 76 | 830 | 22.68 (0.00 – 9,914.43) |
| - Minor conflict | 99 | 686 | 1.96 (0.01 – 42.22) | - | - |  |
| - War | 47 | 332 | 53.68 (0.11 – 9,914.43) | - | - |  |

**Figure S1. Inspection of mortality data showing sudden shifts in mortality in consecutive years**


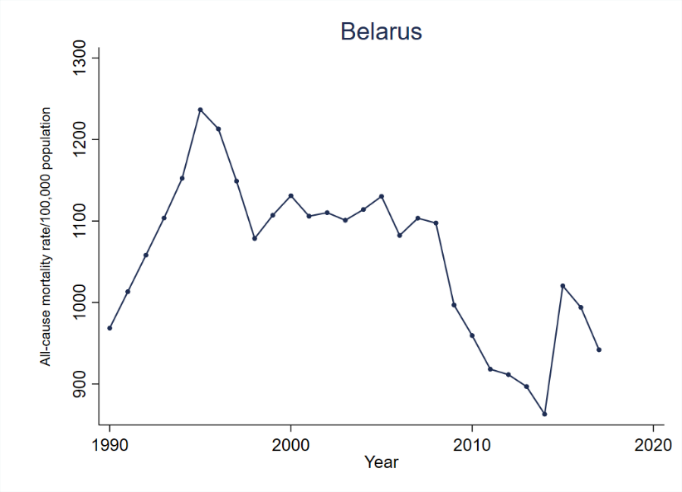

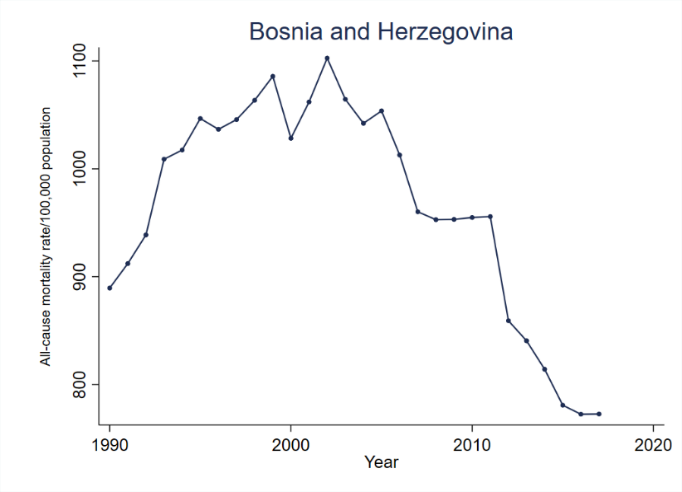

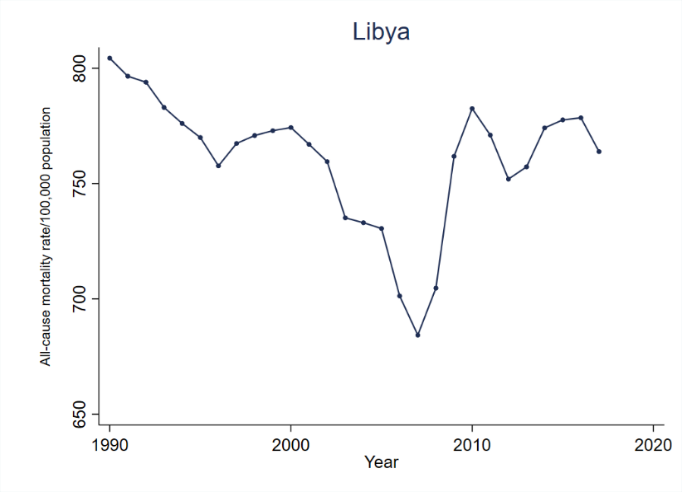

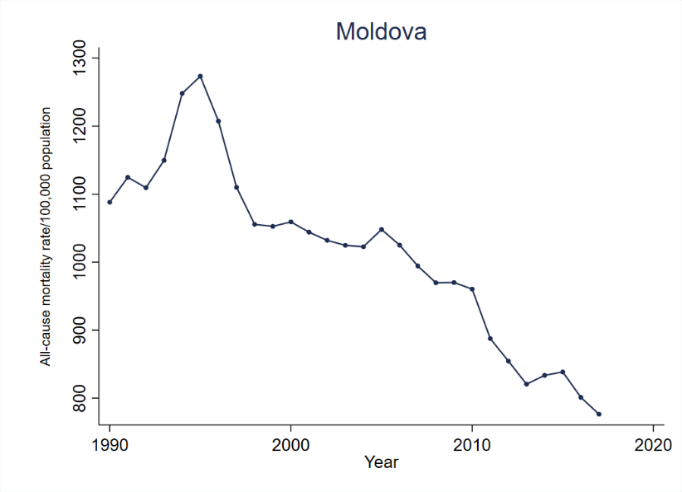

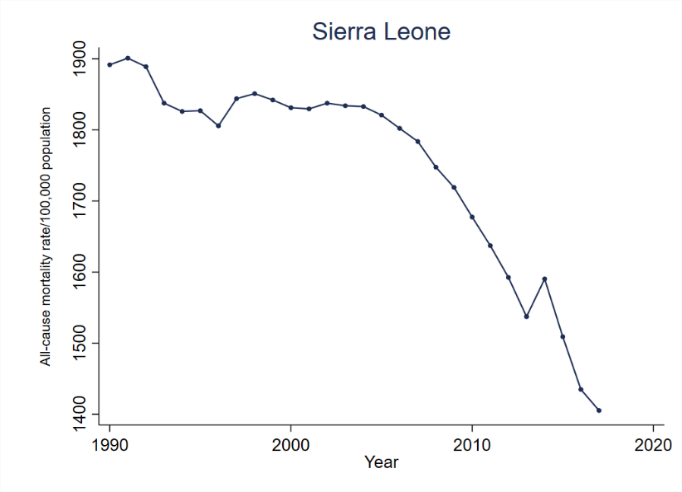

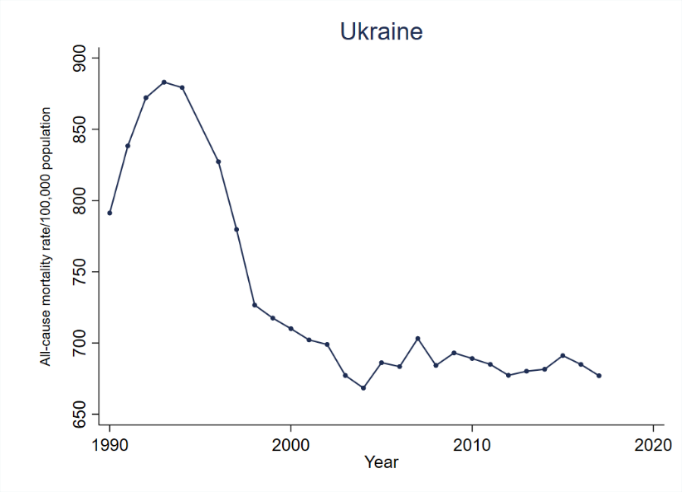


**References**

1. Uppsala Universitet. Department of Peace and Conflict Research [online]. Available at: <https://bit.ly/2Xant85> [Date of access 27 June 2019].

2. Center for Systemic Peace. INSCR Data Page [online]. Available at: <https://bit.ly/1RD6fl7> [Date of access 12 April 2019].

3. Marshall MG. Major episodes of political violence (MEPV) and conflict regions, 1946-2015. *Center for Systemic Peace* 2016; **25**.
