# Supplementary material for: Estimating indirect mortality impacts of armed conflict in civilian populations: panel regression analyses of 193 countries, 1990–2017
Source: BMC Med. 2020 Sep 10;18:266. doi: 10.1186/s12916-020-01708-5 (PMC7487992; doi:10.1186/s12916-020-01708-5)
Supplement: Supplementary file 2 — Additional file 2. Conceptual framework. [file 12916_2020_1708_MOESM2_ESM.docx]

**ADDITIONAL FILE 2. CONCEPTUAL FRAMEWORK**

While armed conflict is clearly antithetic to public health, complex and overlapping mechanisms underpin this causal pathway. Prior conceptualisations are framed by assertions that public health is mainly determined by health spending, and therefore it is health spending through which armed conflict exerts its effects on population health.^1-3^ This has major limitations since health systems is only one of many important determinants of public health.^4^ We attempt to simplify seven micro- to macro-causal pathways through which armed conflict affects civilian health (Figure), and is built on prior frameworks from the literature.^2,5-7^ These pathways are not mutually exclusive, and in reality will co-exist, reinforce one another, and be context-dependent, but they are designed such that each pathway is not contingent on another and can, in theory, exert independent effects. From a statistical perspective, variables along these seven pathways do not require adjustment in any models as they form part of the effect that is being captured.

The most salient way through which armed conflict negatively affects civilian health is by being caught in the line of fire. Over the last 100 years or the casualties from armed conflict has shifted from 90% being soldiers to 90% being civilians.^8^ We term this the **military pathway.** Those who are not killed immediately by bullets, explosions, missiles, toxic fumes, and collateral damage, may die from wounds and injuries in the days and weeks that follow. Others may sustain lifelong disabilities following amputation or injury. During the 1994 Rwanda genocide, civilians with assets were more likely to be targeted for murder than those without.^9^ In the same armed conflict, HIV-infected Hutu men would deliberately rape women to inflict an agonising death from AIDs.^10^ Victims of siege may slowly starve to death and survivors, especially infants and adolescents, may have permanent alternations to their metabolism and growth.^11-13^ Physical wounds are, however, the tip of the iceberg when contrasted to mental wounds that predominate from seeing and experiencing the graphic nature of armed conflict, including rape, torture, execution, and massacre.

The risk of poor mental health resulting from armed conflict is likely to extend beyond those who are in the direct line of fire. Hence, the **mental health pathway** is distinct in that it encompasses all causes of anxiety, stress, and psychological trauma linked to armed conflict. Armed conflict survivors have often lost family members, friends, livelihoods, and identity. Living in a refugee camp, and the stigma associated with being a refugee, can create secondary psychological effects. Chronic stress may increase hypertension risk^14^ and generate susceptibility to other diseases through changes in hormonal balances and the immune response.^15^ Furthermore, poor mental health and wellbeing may place civilians more at risk of using coping mechanisms that are bad for physical and mental health, such as tobacco and alcohol use, inadequate nutrition, and sedentary living.^16^

Civilians are likely to flee their homes if they are able to do so, whether they have experienced the graphic nature of armed conflict or simply perceive it as an imminent threat. Young men may flee in droves to avoid recruitment by warring parties. Military objectives may also target civilians in order to loot their resources or to reduce the fighting efficiency of the enemy (e.g. having less places to hide, gaining less support, etc).^17^ We call this the **forced displacement pathway**. Movement of people often means movement of infections, especially if civilians traverse unfamiliar rural areas, forests, and dumps where the incidence of infections is high and then settle with unexposed or unimmunised host communities. For example, malaria was the primary cause of mortality among Cambodian refugees that arrived to Eastern Thailand in 1979.^18^ Furthermore, overcrowded housing, either in humanitarian refugee camps or in neighbouring towns and cities, is a common feature among the forcibly displaced, which lends itself to poor hygiene, poor sanitation and the further spread of infections. The loss of earnings and assets caused by forced displacement reduces the affordability of essential goods and services, including food and healthcare, which taken together with an increase in competition for local resources can cause soaring prices, food insecurity and famine. A household survey in Uganda found that two thirds of respondents had lost all their assets following bombings, looting, and theft of cattle.^19^ Among the forcibly displaced will be healthcare professionals who would have left clinics and hospitals understaffed and, therefore, unavailable for those remaining behind in need of them. Following the US-led invasion of Iraq in 2003, for example, most Iraqi doctors were believed to have fled the country.^20^

Armed conflict tears of fabric of society, erodes social trust, and generates feelings of animosity, social injustice, and revenge. This includes societies of states that have declared war but do not experience fighting on their land. The breakdown of society that changes socio-cultural norms may increase violence, including gender-based violence, and these are captured by the **social cohesion pathway**. This was most palpably seen in the 1994 Rwanda genocide, where Hutu would not only kill Tutsi unknown to them, but also their neighbours and family members, eliminating all social capital.^21^ In another example, immediately following the Korean and Vietnam wars, Gerosi and King found increased rates of homicides, suicides, transportation deaths and other unintentional injuries the United States.^22^ The embittered situation is made worse given the wide availability of small arms and light weapons both during and after armed conflicts.

The **damaged infrastructure pathway** represented the tactics of armed conflict that involve the targeting essential infrastructures in a bid to disrupt economic capacity, hamper enemy supplies, cause anarchy, and force surrender, but these actions greatly impact civilian health. Blockaded roads and interruptions to transport services prevent supplies of essential goods and services and constrain attempts by civilians to access healthcare and employment. Damage to agricultural production, such as deliberate crop field burning, result in food shortages, malnutrition and the risk of famine. Damaged water and sewage treatment plants can result in widespread contamination that can breed infection. Hospitals may be targeted deliberately or caught in collateral damage, further reducing the availability of health services. Taken together such damage causes a double loss to society: the loss from what resources they were previously contributing and the loss from the damage they now inflict.^17^

Weapons used in armed conflicts may contaminate the surrounding environment and further afield for years after ceasefire. We term this the **environmental toxicification pathway**. Contamination may be following to the release of heavy metals and other substances into the atmosphere during active fire, the seeping of degrading materials into the soil and water supplies from abandoned tanks and artillery shells, and the use of landmines in retreating and surrendering parties. Despite fighting in Cambodia having stopped in 1991, it still remains one of the most heavily landmined areas of the world, killing on average two people per day and restricting access to homes, hospitals, agricultural land, water sources, markets, and even neighbouring villages.^17^

Armed conflict is an expensive operation that invariably involves the diversion of public resources to support military and defence objectives. Civilians fearing the security of their finances may also shift private wealth abroad. The **economic pathway** describes the macro-opportunity cost of war, that is, public spending that could have gone on education, welfare, and healthcare, and the private savings that could have stayed in the country, had armed conflict not occurred. One analysis suggests that military expenditure as a percentage of GDP rises on average from 2.8% to 5.0% during armed conflict.^23^ A weakened economy means less tax revenue and lower healthcare spending, which is likely to result in unpaid or grossly reduced healthcare worker salaries, less available health services for those in need, de-prioritisation of primary prevention services such as screening, and less regulation of healthcare in general. The ability of the state to respond to emerging threats to public health will be weakened, resulting in a continual growth of poor population health. Reduced private sector spending on health often follows. Even in non-state armed conflicts, military parties that engage in war require large amounts of resource that could have been used on local health-promoting initiatives. Empirical research suggests an additional 2.2% of GDP spent on the military would lead to permanent loss of around 2% of GDP over the course of a typical seven year conflict.^23^ Furthermore, military expenditure as a percentage of GDP does not tend to return to pre-war levels in the aftermath of armed conflict, rather it falls on average by only 0.5% during ten years of post-conflict peace.^23^ Capital flight continues to occur in the post-conflict period; estimates suggest that on average 26.1% of private wealth is moved abroad by the first decade of post-conflict peace.^17^

**Drivers of armed conflict and health**

The complex relationship between armed conflict and health could be distorted by the presence of other, confounding factors. Different factors may determine different aspects of armed conflict (e.g. its initiation, continuation, intensity, and cessation), and the drivers of armed conflict are as varied as the societies that produce them. We undertook an extensive literature review to identify seven key factors, which we include on our conceptual framework (Figure 1) and describe below. Unlike variables that lie on the causal pathway above, drivers of armed conflict and health require inclusion and adjustment in any model specifications.

*1. National wealth*

Lower national wealth means a smaller pool of resource to spend on public health, preventive and curative services, and other welfare and social services that are health promoting. Low per capita income also means less of the population is able to afford healthcare and other essential services that determine health. There are also detrimental psychological, social, and financial effects on individuals in times of economic recession.^24^ National poverty, in the sense of low rates of economic growth and a low level of national development, and some argue that it the most robust predictor for armed conflict incidence whose effect is often understated.^17^ That is not to say that high-income countries are immune from armed conflict, as shown by previous violence in Northern Ireland and the Pays Basque region of Spain, but the threat of armed conflict is greater in low-income countries. In particular, the combination of low, declining, and unequally distributed incomes can create a pool of impoverished and disaffected young men who can be cheaply recruited by warring parties.^17^ Countries where natural resources provide a source of wealth may encourage the local population to support political demands for secession.^17^ Finally, it is important to consider the fact that the relationship between national wealth and health may be bidirectional; a healthier population is more able to work and contribute to the national economy.

2. *Political system*

Democratic countries tend to have better health and lower mortality than autocratic ones as they are more responsive to the needs of their population and less likely to divert public resources to the military in order to preserve power.^25^ Income distributions are more equitable and per capita income grows faster in democracies. Political leaders in democracies retain power by investing widely in public services, including health services, in order to satisfy the population majority.^26^ Other mechanisms include increased accountability, transparency and protection of the media, all of which can feed back into political decisions to improve public health.^26^ This alludes to the common argument that health differences between the two political systems are essentially due to differences in health expenditure. However, strongly autocratic countries such as Qatar and Saudi Arabia have a high GDP per capita while maintaining good population health akin to high-income democratic countries.^25^ Empirical research has shown that partial democracies are more prone to armed conflict than democracies and autocracies, which are as safe as each other.^27^ This could be explained by the fact that partial democracies allows some opposition but does not give them any real influence, or by confounding by national wealth i.e. partial democracies tend to have lower GDP per capita than other political systems. At high GDP per capita, democracies are protective against armed conflict but this is not necessarily true for low-income countries.^17^

*3. Population density and urban growth*

Countries and cities with a high population density are associated with an increased risk of infection transmission, are more noisy and polluted, have more road traffic accidents, and are associated with stress, smoking, and all-cause mortality.^28^ Despite this, coverage of healthcare is more feasible in dense rather than dispersed populations,^29^ and higher density areas, particularly in high income settings, is associated with higher levels of physical activity.^30^ Urban areas that experience rapid growth can a result in a surge in the demand for healthcare, but health systems often lag behind meeting this extra demand with increased supply. Rapid urban growth may be the consequence of armed conflict in a neighbouring country creating an influx of migration, or following internal migration from rural areas. The latter often happens when agricultural workers suffer at the hands of climate change and are seeking new opportunities and employment in the country’s major cities. When rapid urban growth occurs, disease surveillance, immunisation coverage, and the provision of safe water become more difficult. New city dwellers may resort to living in slums and are often underrepresented in political processes, including the processes that offer equitable and fair resource allocation for education, employment, and health. This may lead to social unrest and armed conflict.

*4. Education*

Educational attainment is a key determinant of health as health literate populations have greater awareness of risks to their health and can make better and more efficient use of public health resources available to them (e.g. screening and vaccination uptake, self-care, etc). Education also exerts its effects on health through national wealth; educated societies can contribute productively to the economy by virtue of being in stable, permanent employment. Educated women may delay pregnancy or have fewer children, thereby increasing the number employment years available to them. Educational attainment is also a marker for national development, which by some scholars is considered a stronger driver for armed conflict than national wealth.^31^

*5. Income inequality*

Absolute poverty makes healthcare and other health-generating services unaffordable. However, the relative difference between the rich and poor, income inequality, also determines health by eroding social capital. A high income inequality society means a greater proportion of the population is in the low income category and hence below the poverty line. Lower income groups are less likely to be effectively represented in the political competition for scarce healthcare resources. Wealthier groups are able to dominate the political system for their own health benefit and for the benefit of the privileged and powerful, and often receive better care and lower marginal utility than poorer segments of the population.^1^ The two components of income inequality, absolute and relative poverty, together can be a powerful driver for social and political unrest.^17^

*6. Ethnic fractionalisation*

Longstanding ethnically or religiously heterogeneous societies may generate cleavages that result in discriminatory access to health and welfare services. For example, Lebanon’s main political particles (the Shi’a Hezbollah and the Sunni Future Movement) run large networks of health clinics, dispensaries, hospitals, schools, and social assistance programs, preferential treatment of which is given to those of the same religious sect.^32^ These same ethnic or religious biases are considered by many as a basis for social unrest that could result in armed conflict.^17^ However, the issue is complicated by the number and size of different ethnic groups; research suggests that substantial ethnic and religious diversity is actually protective against armed conflict as nationwide collective action becomes more difficult.^33^ The risk of armed conflict is therefore higher in ethnically homogeneous societies, in societies where the largest ethnic group has an absolute ruling majority, and in polarised societies divided into two equal groups.^33^ Natural resources are rarely uniformly distributed over an entire country, and the local population that discovers this resource may claim ownership and use it to fuel secession.^17^

*7. Climate*

Threats from climate change have long been recognised as a global health concern. Some countries are endemic to infectious diseases such as malaria, tuberculosis and diarrhoeal diseases, where vectors are able to flourish in the existing and often tropical climate. Degradation of arid or semi-arid land, diminished water availability, heatwaves, and drought may debilitate the agricultural sector, resulting in high food prices, food insecurity, high rural unemployment, social unrest, and migration to urban cities to seek new opportunities. Such examples were seen in the recent civil wars of Syria and Sudan.^34^ Tensions between countries may rise if dams are built to respond to water shortages, as is seen in Iraq and among Kurds following Turkey’s construction of the Ilisu Dam.^35^ Sudden climate change-related shocks, such as natural disasters and drought, can destabilise supply chains and adversely affect economies, precipitating armed conflict.

**Figure S2.** Conceptual framework of armed conflict and health


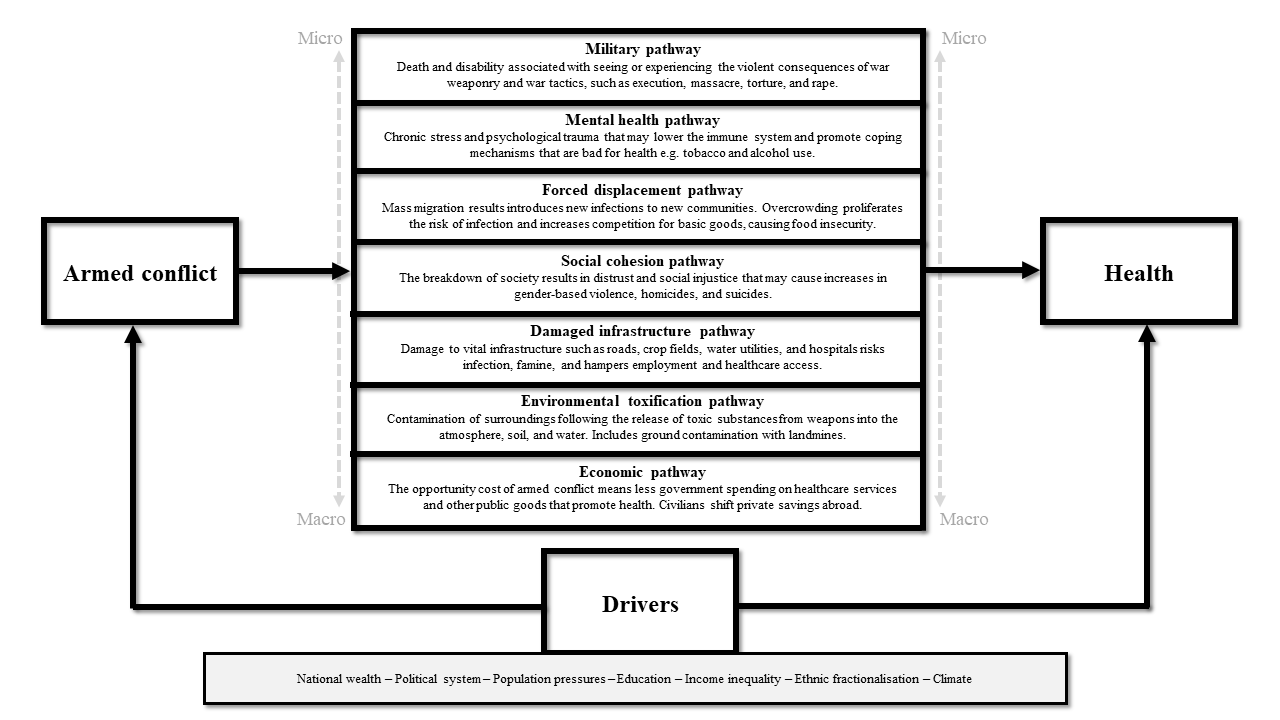


**References**

1. Ghobarah HA, Huth P, Russett B. Civil wars kill and maim people—long after the shooting stops. *Am Polit Sci Rev* 2003; **97**(2): 189-202.

2. Ghobarah HA, Huth P, Russett B. Comparative public health: The political economy of human misery and Well‐Being. *Int Stud Quart* 2004; **48**(1): 73-94.

3. Ghobarah HA, Huth P, Russett B. The post-war public health effects of civil conflict. *Soc Sci Med* 2004; **59**(4): 869-84.

4. Marmot M, Friel S, Bell R, Houweling TAJ, Taylor S. Closing the gap in a generation: health equity through action on the social determinants of health. *Lancet* 2008; **372**(9650): 1661-9.

5. Iqbal Z, Zorn C. Violent conflict and the spread of HIV/AIDS in Africa. *J Polilt* 2010; **72**(1): 149-62.

6. Li Q, Wen M. The immediate and lingering effects of armed conflict on adult mortality: a time-series cross-national analysis. *J Peace Res* 2005; **42**(4): 471-92.

7. Plümper T, Neumayer E. The unequal burden of war: The effect of armed conflict on the gender gap in life expectancy. *Int Organ* 2006; **60**(3): 723-54.

8. Cairns E. A safer future: Reducing the human cost of war: Oxfam GB; 1997.

9. André C, Platteau J-P. Land relations under unbearable stress: Rwanda caught in the Malthusian trap. *J Econ Behav Organ* 1998; **34**(1): 1-47.

10. Elbe S. HIV/AIDS and the Changing Landscape of War in Africa. *Int Security* 2002; **27**(2): 159-77.

11. Koupil I, Plavinskaja S, Parfenova N, Shestov DB, Danziger PD, Vagero D. Cancer mortality in women and men who survived the siege of Leningrad (1941-1944). *Int J Cancer* 2009; **124**(6): 1416-21.

12. Stanner SA, Bulmer K, Andres C, et al. Does malnutrition in utero determine diabetes and coronary heart disease in adulthood? Results from the Leningrad siege study, a cross sectional study. *BMJ* 1997; **315**(7119): 1342-8.

13. Vågerö D, Koupil I, Parfenova N, Sparen P. Long term health consequences following the Siege of Leningrad; 2013.

14. Sparrenberger F, Cichelero FT, Ascoli AM, et al. Does psychosocial stress cause hypertension? A systematic review of observational studies. *J Hum Hypertens* 2009; **23**(1): 12-9.

15. Padgett DA, Glaser R. How stress influences the immune response. *Trends Immunol* 2003; **24**(8): 444-8.

16. Jawad M, Vamos EP, Najim M, Roberts B, Millett C. Impact of armed conflict on cardiovascular disease risk: a systematic review. *Heart (British Cardiac Society)* 2019.

17. Collier P. Breaking the conflict trap: Civil war and development policy: World Bank Publications; 2003.

18. Glass RI, Cates W, Jr., Nieburg P, et al. Rapid assessment of health status and preventive-medicine needs of newly arrived Kampuchean refugees, Sa Kaeo, Thailand. *Lancet* 1980; **1**(8173): 868-72.

19. Matowu J, Stewart F, Fitzgerald V. Uganda: the social and economic costs of conflict. *War and Underdevelopment* 2001; **2**: 240-303.

20. Al Hilfi TK, Lafta R, Burnham G. Health services in Iraq. *Lancet* 2013; **381**(9870): 939-48.

21. Colletta NJ, Cullen ML. Violent conflict and the transformation of social capital: Lessons from Cambodia, Rwanda, Guatemala, and Somalia: World Bank Publications; 2000.

22. Gerosi F, King G. Short Term Effects of War Deaths on Public Health in the US. Cambridge, MA: Harvard Center for Basic Research in the Social Sciences, working paper

2002.

23. Loayza N, Knight M, Villanueva D. The peace dividend: military spending cuts and economic growth: The World Bank; 1999.

24. Frasquilho D, Matos MG, Salonna F, et al. Mental health outcomes in times of economic recession: a systematic literature review. *BMC Public Health* 2015; **16**(1): 115.

25. Iqbal Z. Health and human security: The public health impact of violent conflict. *Int Stud Quart* 2006; **50**(3): 631-49.

26. Bollyky TJ, Templin T, Cohen M, Schoder D, Dieleman JL, Wigley S. The relationships between democratic experience, adult health, and cause-specific mortality in 170 countries between 1980 and 2016: an observational analysis. *Lancet* 2019; **393**(10181): 1628-40.

27. Fearon JD, Laitin DD. Ethnicity, insurgency, and civil war. *Am Polit Sci Rev* 2003; **97**(1): 75-90.

28. Beenackers MA, Oude Groeniger J, Kamphuis CBM, Van Lenthe FJ. Urban population density and mortality in a compact Dutch city: 23-year follow-up of the Dutch GLOBE study. *Health Place* 2018; **53**: 79-85.

29. Hanlon M, Burstein R, Masters SH, Zhang R. Exploring the relationship between population density and maternal health coverage. *BMC health services research* 2012; **12**: 416.

30. Barnett DW, Barnett A, Nathan A, et al. Built environmental correlates of older adults’ total physical activity and walking: a systematic review and meta-analysis. *Int J Behav Nutr Phys Act* 2017; **14**(1): 103.

31. Sambanis N. Do ethnic and nonethnic civil wars have the same causes? A theoretical and empirical inquiry (Part 1). *J Conflict Resolut* 2001; **45**(3): 259-82.

32. Cammett M. Sectarianism and the Ambiguities of Welfare in Lebanon. *Curr Anthropol* 2015; **56**(S11): S76-S87.

33. Collier P, Hoeffler A. Greed and grievance in civil war. *Oxford Econ Pap* 2004; **56**(4): 563-95.

34. Bowles DC, Butler CD, Morisetti N. Climate change, conflict and health. *J R Soc Med* 2015; **108**(10): 390-5.

35. Hommes L, Boelens R, Maat H. Contested hydrosocial territories and disputed water governance: Struggles and competing claims over the Ilisu Dam development in southeastern Turkey. *Geoforum* 2016; **71**: 9-20.
