# Supplementary material for: Estimating indirect mortality impacts of armed conflict in civilian populations: panel regression analyses of 193 countries, 1990–2017
Source: BMC Med. 2020 Sep 10;18:266. doi: 10.1186/s12916-020-01708-5 (PMC7487992; doi:10.1186/s12916-020-01708-5)
Supplement: Supplementary file 3 — Additional file 3. First order causes of death. [file 12916_2020_1708_MOESM3_ESM.docx]

**ADDITIONAL FILE 3. FIRST ORDER CAUSES OF DEATH**

**Table S3.** **The association between armed conflict and age-standardised cause-specific mortality, 1990-2017**

|  | **Communicable, maternal, nutritional, and neonatal diseases** | **Non-communicable diseases** | **Injuries** |
| --- | --- | --- | --- |
| **Armed conflict exposure** | β (95% CI) | β (95% CI) | β (95% CI) |
| None (<25 battle deaths/year) | 0.00 | 0.00 | 0.00 |
| Minor conflict (25-999 battle deaths/year) | 15.17 (-10.76, 41.10) | -1.96 (-14.24, 10.32) | -0.16 (-3.98, 3.66) |
| War (≥1,000 battle deaths/year) | 51.25 (2.60, 99.89) * | 22.71 (0.23, 45.19) * | 7.56 (3.43, 11.70) *** |
|  |  |  |  |
| **Covariates** |  |  |  |
| GDP per capita | 0.00 (0.00, 0.01) *** | -0.00 (-0.00, -0.00) *** | -0.00 (-0.00, 0.00) |
| OECD membership | 38.20 (8.15, 68.25) * | -60.52 (-98.10, -22.94) ** | -16.72 (-27.69, -5.75) ** |
| Population density | -64.73 (-270.5, 141.0) | 36.40 (-116.2, 189.0) | 8.76 (-16.50, 34.02) |
| Urbanisation | -11.95 (-21.79, -2.11) * | -3.38 (-8.28, 1.51) | -1.12 (-2.09, -0.15) * |
| Age dependency ratio | -2.01 (-4.03, 0.01) | -0.44 (-1.77, 0.89) | -0.10 (-0.29, 0.10) |
| Male education | -13.06 (-47.35, 21.23) | -17.34 (-46.97, 12.28) | -3.34 (-7.74, 1.06) |
| Temperature | 2.84 (-4.58, 10.25) | -9.31 (-22.52, 3.90) | -0.84 (-1.78, 0.10) |
| Rainfall | 17.42 (-9.54, 44.37) | -2.08 (-13.93, 9.77) | 7.38 (-3.30, 18.06) |
| Earthquake | -2.95 (-8.87, 2.98) | -3.32 (-11.59, 4.96) | 11.97 (-6.78, 30.72) |
| Drought | -0.98 (-12.10, 10.13) | 3.27 (-2.13, 8.88) | -1.20 (-3.77, 1.38) |
| Observations | 4,754 | 4,754 | 4,754 |
| Countries | 183 | 183 | 183 |

**Note:** ^*^ *p* < 0.05, ^**^ *p* < 0.01, ^***^ *p* < 0.001. Robust standard errors were employed. Each column is the output from one panel regression with fixed effects adjusted for the covariates in the table in addition to year dummies (not shown). Coefficients are interpreted as the change in all-cause mortality per 100,000 following a change in one unit of the independent variable.

GDP per capita is in current US dollars. Population density represents the percentage of the population living in a density of >1,000 ppl/sqkm. Urbanisation represents the percentage of the population living in urban areas. The age dependency ratio represents the percentage of the population younger than 15 years and older than 64 years per 100 working-age population. Male education is expressed as years per capita and is age-standardised. Temperature is in degrees Celsius and is the mean population-weighted annual temperature. Rainfall represents the percentage of the population living in the top world quintile of annual rainfall. Earthquake and drought are binary variables representing their absence or presence. All armed conflict variables were lagged by one year.
