# Supplementary material for: Estimating indirect mortality impacts of armed conflict in civilian populations: panel regression analyses of 193 countries, 1990–2017
Source: BMC Med. 2020 Sep 10;18:266. doi: 10.1186/s12916-020-01708-5 (PMC7487992; doi:10.1186/s12916-020-01708-5)
Supplement: Supplementary file 4 — Additional file 4. Second order causes of death. [file 12916_2020_1708_MOESM4_ESM.docx]

**ADDITIONAL FILE 4. SECOND ORDER CAUSES OF DEATH**

**Figure S4. The association between war and first and second order causes of death**

Injuries

Non-communicable diseases

Communicable, maternal, neonatal, and nutritional diseases
