# Supplementary material for: Estimating indirect mortality impacts of armed conflict in civilian populations: panel regression analyses of 193 countries, 1990–2017
Source: BMC Med. 2020 Sep 10;18:266. doi: 10.1186/s12916-020-01708-5 (PMC7487992; doi:10.1186/s12916-020-01708-5)
Supplement: Supplementary file 7 — Additional file 7. Lags. [file 12916_2020_1708_MOESM7_ESM.docx]

**ADDITIONAL FILE 7. LAGS**

**Figure S7.** **Lagged effects showing the association between war and age-standardised all-cause and cause-specific mortality, 1990-2017**

**Caption**: Each line is the coefficient and 95% confidence interval of the war variable, lagged at between one and ten years. Each line is the from the output of a fixed effects panel regression model adjusting for a categorical time dummy, GDP per capita, OECD membership, population density, urban residence, age dependency ratio, male education, temperature, rainfall and the presence of earthquakes and droughts. Coefficients are interpreted as the absolute change in mortality following the onset of war.
