# Supplementary material for: Estimating indirect mortality impacts of armed conflict in civilian populations: panel regression analyses of 193 countries, 1990–2017
Source: BMC Med. 2020 Sep 10;18:266. doi: 10.1186/s12916-020-01708-5 (PMC7487992; doi:10.1186/s12916-020-01708-5)
Supplement: Supplementary file 8 — Additional file 8. Differences by actor type. [file 12916_2020_1708_MOESM8_ESM.docx]

**ADDITIONAL FILE 8. DIFFERENCES BY ARMED CONFLICT TYPE**

**Table S8.1. Sample description by armed conflict type**

|  | State-based conflicts | | | | Non-state conflicts | | | | One-sided violence | | | |
| --- | --- | --- | --- | --- | --- | --- | --- | --- | --- | --- | --- | --- |
|  | Countries (N) | Observations (N) | Battle-related deaths per 100,000 population (mean, SD) | Civilian mortality rate per 100,000 population (mean, SD) | Countries (N) | Observations (N) | Battle-related deaths per 100,000 population (mean, SD) | Civilian mortality rate per 100,000 population (mean, SD) | Countries (N) | Observations (N) | Battle-related deaths per 100,000 population (mean, SD) | Civilian mortality rate per 100,000 population (mean, SD) |
| Armed conflict exposure^1^ | |  |  |  |  |  |  |  |  |  |  |  |
| No | 188 | 4,613 | 0.01 (0.06) | 977.40 (466.27) | 192 | 4,986 | 0.003 (0.07) | 994.99 (478.19) | 189 | 4,790 | 0.004 (0.03) | 983.01 (468.05) |
| Yes | 83 | 791 | 8.05 (27.27) | 1,274.14 (511.11) | 60 | 418 | 2.03 (6.66) | 1,329.11 (452.86) | 80 | 586 | 21.06 (408.19) | 1,330.66 (506.10) |
| - Minor conflict | 81 | 562 | 2.05 (4.07) | 1,267.37 (516.27_ | 59 | 379 | 1.06 (2.02) | 1,335.18 (443.08) | 79 | 533 | 1.34 (2.68) | 1,310.84 (494.89) |
| - War | 39 | 229 | 22.76 (47.23) | 1,290.75 (498.93) | 15 | 39 | 11.42 (18.60) | 1,270.15 (541.96) | 18 | 53 | 220.11 (1,355.13) | 1,530.70 (575.70) |
|  |  |  |  |  |  |  |  |  |  |  |  |  |
| Quintile of exposure^2^ | |  |  |  |  |  |  |  |  |  |  |  |
| - None | 184 | 4,362 | - | 970.38 (464.19) | 192 | 4,807 | - | 988.40 (478.49) | 188 | 4,427 | - | 960.31 (456.31) |
| - First | 54 | 209 | 0.02 (0.02) | 978.97 (391.75) | 30 | 120 | 0.008 (0.01) | 1,091.16 (336.50) | 48 | 196 | 0.007 (0.005) | 1,101.76 (431.23) |
| - Second | 60 | 208 | 0.14 (0.06) | 1,162.31 (437.59) | 41 | 119 | 0.06 (0.03) | 1,232.55 (429.13) | 52 | 195 | 0.04 (0.01) | 1,181.13 (436.09) |
| - Third | 51 | 209 | 0.57 (0.23) | 1,229.98 (493.30) | 39 | 120 | 0.24 (0.07) | 1,314.33 (394.21) | 59 | 196 | 0.14 (0.05) | 1,256.36 (499.05) |
| - Fourth | 54 | 208 | 2.48 (1.10) | 1,313.37 (524.58) | 39 | 119 | 0.70 (0.22) | 1,329.96 (473.79) | 58 | 195 | 0.55 (0.21) | 1,364.03 (474.83) |
| - Fifth | 45 | 208 | 27.50 (48.15) | 1,476.99 (555.29) | 28 | 119 | 6.26 (11.46) | 1,443.65 (536.55) | 46 | 195 | 62.85 (708.18) | 1,573.45 (581.45) |

^1^Based on the number of battle-related deaths per country-conflict-year: No: <25, Yes: ≥25, Minor conflict: 25-999, War: ≥1,000; ^2^Based on the number of battle-related deaths per 100,000 population per country-year.

**Table S8.2. The association between armed conflict type and age-standardised all-cause mortality, 1990-2017 (beta coefficients, 95% CIs)**

|  | **Model 1** | **Model 2** |
| --- | --- | --- |
| **Armed conflict type** |  |  |
| No conflict | - | 0.00 |
| State-based conflict | - | 19.82 (-12.64, 52.27) |
| Non-state conflict | - | -29.92 (-63.46, 3.62) |
| One-sided violence | - | -9.54 (-48.11, 29.03) |
|  |  |  |
| **Battle-related deaths/100, 000 population** | 4.21 (-2.33, 10.75) | 4.29 (-2.32, 10.91) |
|  |  |  |
| **Interaction** |  |  |
| No conflict x Battle-related deaths/100,000 population | 0.00 | 0.00 |
| State-based x Battle-related deaths/100,000 population | -2.19 (-8.79, 4.42) | -3.03 (-10.07, 4.01) |
| Non-state x Battle-related deaths/100,000 population | -2.61 (-15.36, 10.14) | 0.57 (-13.52, 14.66) |
| One-sided violence x Battle-related deaths/100,000 population | -39.04 (-84.44, 6.35) | -31.81 (-89.28, 25.68) |
|  |  |  |
| **Covariates** |  |  |
| GDP per capita | -0.00 (-0.00, 0.00) | -0.00 (-0.00, 0.00) |
| OECD membership | -51.44 (-108.6, 5.73) | -51.47 (-108.7, 5.73) |
| Population density | -81.81 (-404.3, 240.7) | -84.20 (-407.9, 239.5) |
| Urbanisation | -11.25 (-25.90, 3.41) | -11.23 (-25.88, 3.42) |
| Age dependency ratio | -2.11 (-5.17, 0.96) | -2.12 (-5.18, 0.95) |
| Male education | -51.63 (-102.5, -0.76) * | -51.77 (-102.6, -0.90) |
| Temperature | -8.91 (-27.39, 9.57) | -8.89 (-27.45, 9.66) |
| Rainfall | 27.89 (-4.23, 60.00) | 28.02 (-3.97, 60.01) |
| Earthquake | 5.49 (-20.40, 31.38) | 5.40 (-20.45, 31.25) |
| Drought | 3.41 (-10.29, 17.11) | 3.26 (-10.44, 16.95) |
| Observations | 4,133 | 4,133 |
| Countries | 176 | 176 |

**Note:** ^*^ *p* < 0.05, ^**^ *p* < 0.01, ^***^ *p* < 0.001. Robust standard errors were employed. Each column is the output from one panel regression with fixed effects adjusted for the covariates in the table in addition to categorical year dummies (not shown). Coefficients are interpreted as the change in all-cause mortality per 100,000 following a change in one unit of the independent variable.

GDP per capita is in current US dollars. Population density represents the percentage of the population living in a density of >1,000 ppl/sqkm. Urbanisation represents the percentage of the population living in urban areas. The age dependency ratio represents the percentage of the population younger than 15 years and older than 64 years per 100 working-age population. Male education is expressed as years per capita and is age-standardised. Temperature is in degrees Celsius and is the mean population-weighted annual temperature. Rainfall represents the percentage of the population living in the top world quintile of annual rainfall. Earthquake and drought are binary variables representing their absence or presence. All armed conflict variables were lagged by one year.

**Figure S8.1. Scatter plots correlating the rate of battle-related deaths with the age-standardised mortality rate by armed conflict type (restricted to observations where the rate of battle-related deaths>0)**
