# Supplementary material for: Estimating indirect mortality impacts of armed conflict in civilian populations: panel regression analyses of 193 countries, 1990–2017
Source: BMC Med. 2020 Sep 10;18:266. doi: 10.1186/s12916-020-01708-5 (PMC7487992; doi:10.1186/s12916-020-01708-5)
Supplement: Supplementary file 9 — Additional file 9. Sensitivity analysis. [file 12916_2020_1708_MOESM9_ESM.docx]

**ADDITIONAL FILE 9. SENSITIVITY ANALYSES**

**Table S9.1. The association between armed conflict and age-standardised all-cause mortality with varying model specifications, 1990-2017 (beta coefficients, 95% CIs)**

|  | **Random effects** | **Fixed effects (without clustering)** | **Fixed effects (with clustering)** |
| --- | --- | --- | --- |
| **Armed conflict exposure** |  |  |  |
| None (<25 battle deaths/year) | 0.00 | 0.00 | 0.00 |
| Minor conflict (25-999 battle deaths/year) | 18.23* (3.34, 33.13) | 16.27* (1.36, 31.17) | 16.27 (-21.41, 53.94) |
| War (≥1,000 battle deaths/year) | 77.35*** (55.70, 99.01) | 74.59*** (52.92, 96.26) | 74.59* (-0.34, 148.8) |
| Observations | 5,211 | 5,211 | 5,211 |
| Countries | 193 | 193 | 193 |

**Note:** ^*^ *p* < 0.05, ^**^ *p* < 0.01, ^***^ *p* < 0.001. Each column is the output from one panel regression with country-year fixed effects. Coefficients are interpreted as the change in all-cause mortality per 100,000 following a change in one unit of the independent variable. All armed conflict variables were lagged by one year.

**Table S9.2. The association between armed conflict and age-standardised all-cause mortality with sequential addition of covariates, 1990-2017 (beta coefficients, 95% CIs)**

|  | (1) | (2) | (3) | (4) | (5) | (6) | (7) | (8) | (9) | (10) | (11) | (12) |
| --- | --- | --- | --- | --- | --- | --- | --- | --- | --- | --- | --- | --- |
| **Armed conflict exposure** |  |  |  |  |  |  |  |  |  |  |  |  |
| None (<25 battle deaths/year) | 0.00 | 0.00 | 0.00 | 0.00 | 0.00 | 0.00 | 0.00 | 0.00 | 0.00 | 0.00 | 0.00 | 0.00 |
| Minor conflict (25-999 battle deaths/year) | 15.69 (-22.31, 53.68) | 15.98 (-22.02, 53.98) | 15.67 (-22.22, 53.55) | 11.56 (-24.11, 47.23) | 11.54 (-23.81, 46.90) | 12.70 (-22.94, 48.34) | 13.11 (-22.50, 48.73) | 12.95 (-22.66, 48.57) | 13.03 (-22.59, 48.65) | 13.05 (-22.74, 48.84) | 13.73 (-20.76, 48.22) | 18.38 (-21.74, 58.50) |
| War (≥1,000 battle deaths/year) | 93.51* (18.37, 168.6) | 93.97* (18.84, 169.1) | 93.13* (18.48, 167.8) | 81.35* (13.86, 148.8) | 79.84* (12.98, 146.7) | 80.80* (13.68, 147.9) | 81.26* (14.02, 148.5) | 81.23* (13.94, 148.5) | 81.50* (14.35, 148.7) | 81.52* (14.30, 148.7) | 84.16* (17.25, 151.1) | 87.62* (19.14, 156.1) |
|  |  |  |  |  |  |  |  |  |  |  |  |  |
| **Covariates** |  |  |  |  |  |  |  |  |  |  |  |  |
| GDP per capita | 0.00 (-0.00, 0.00) | 0.00 (-0.00, 0.00) | 0.00 (-0.00, 0.00) | 0.00 (-0.00, 0.00) | 0.00 (-0.00, 0.00) | 0.00 (-0.00, 0.00) | 0.00 (-0.00, 0.00) | 0.00 (-0.00, 0.00) | 0.00 (-0.00, 0.00) | 0.00 (-0.00, 0.00) | 0.00 (-0.00, 0.00) | 0.00 (-0.00, 0.00) |
| OECD membership |  | -29.83 (-78.14, 18.48) | -37.06 (-88.86, 14.73) | -41.26 (-97.84, 15.31) | -32.80 (-87.45, 21.86) | -38.54 (-95.71, 18.64) | -38.81 (-96.51, 18.89) | -38.77 (-96.66, 19.11) | -39.06 (-96.75, 18.63) | -39.04 (-96.73, 18.65) | -34.16 (-97.26, 28.94) | -40.45 (-94.34, 13.43) |
| Population density |  |  | -208.5 (-586.6, 169.5) | -61.16 (-400.1, 277.8) | -37.58 (-377.4, 302.2) | -18.03 (-342.4, 306.3) | -20.25 (-344.0, 303.5) | -19.36 (-342.5, 303.8) | -19.53 (-342.7, 303.7) | -19.56 (-342.9, 303.8) | -159.7 (-680.0, 360.7) | -94.22 (-641.9, 453.5) |
| Urbananisation |  |  |  | -16.02* (-29.13, -2.92) | -16.98* (-31.17, -2.78) | -16.75* (-31.05, -2.46) | -16.49* (-30.93, -2.06) | -16.46* (-30.90, -2.03) | -16.46* (-30.90, -2.01) | -16.46* (-30.90, -2.01) | -15.38 (-30.90, 0.13) | -8.57 (-25.72, 8.58) |
| Age dependency ratio |  |  |  |  | -1.94 (-4.89, 1.00) | -2.35 (-5.38, 0.68) | -2.56 (-5.57, 0.46) | -2.55 (-5.57, 0.46) | -2.55 (-5.56, 0.47) | -2.55 (-5.57, 0.47) | -2.63 (-5.95, 0.70) | -4.68* (-9.04, -0.33) |
| Male education |  |  |  |  |  | -32.56 (-85.49, 20.37) | -34.10 (-87.92, 19.71) | -33.74 (-87.43, 19.95) | -33.74 (-87.46, 19.98) | -33.74 (-87.47, 19.98) | -26.72 (-82.59, 29.16) | -21.43 (-105.1, 62.29) |
| Temperature |  |  |  |  |  |  | -7.20 (-24.80, 10.40) | -7.30 (-24.97, 10.37) | -7.30 (-24.96, 10.37) | -7.31 (-24.99, 10.36) | -9.73 (-28.55, 9.09) | -0.03 (-13.71, 13.66) |
| Rainfall |  |  |  |  |  |  |  | 22.53 (-9.99, 55.05) | 22.56 (-9.92, 55.03) | 22.72 (-9.75, 55.18) | 22.17 (-14.25, 58.59) | 28.48 (-3.30, 60.27) |
| Earthquake |  |  |  |  |  |  |  |  | 5.70 (-15.73, 27.13) | 5.71 (-15.72, 27.13) | 4.18 (-17.49, 25.85) | 7.59 (-19.92, 35.09) |
| Drought |  |  |  |  |  |  |  |  |  | 1.19 (-14.13, 16.51) | 0.71 (-15.29, 16.72) | -10.22 (-28.88, 8.44) |
| Multiplicative Polyarchy Index |  |  |  |  |  |  |  |  |  |  | 10.29 (-159.9, 180.5) | 56.23 (-146.7, 259.1) |
| Ethnic Fractionalisation Index |  |  |  |  |  |  |  |  |  |  |  | 343.7 (-650.1, 1337.5) |
| Observations | 4,914 | 4,914 | 4,914 | 4,914 | 4,754 | 4,754 | 4,754 | 4,754 | 4,754 | 4,754 | 4,394 | 3,381 |
| Countries | 190 | 190 | 190 | 190 | 183 | 183 | 183 | 183 | 183 | 183 | 169 | 152 |

**Note:** ^*^ *p* < 0.05, ^**^ *p* < 0.01, ^***^ *p* < 0.001. Robust standard errors were employed. Each column is the output from one panel regression with fixed effects adjusted for the covariates in the table in addition to year dummies (not shown). Coefficients are interpreted as the change in all-cause mortality per 100,000 following a change in one unit of the independent variable.

GDP per capita is in current US dollars. Population density represents the percentage of the population living in a density of >1,000 ppl/sqkm. Urbanisation represents the percentage of the population living in urban areas. The age dependency ratio represents the percentage of the population younger than 15 years and older than 64 years per 100 working-age population. Male education is expressed as years per capita and is age-standardised. Temperature is in degrees Celsius and is the mean population-weighted annual temperature. Rainfall represents the percentage of the population living in the top world quintile of annual rainfall. Earthquake and drought are binary variables representing their absence or presence. The Multiplicative Polyarchy Index represents a proportion between 0 and 1 constructed from five core components of electoral democracy. The Ethnic Fractionalisation Index represents the probability that two randomly drawn individuals within a country are not from the same ethnic group All armed conflict variables were lagged by one year.

**Table S9.3. The association between armed conflict and age-standardised all-cause mortality, 1990-2017 (beta coefficients, 95% CIs)**

|  | **Misclassification bias^1^** | **Major Episodes of Political Violence dataset^2^** |
| --- | --- | --- |
| **Armed conflict exposure** |  |  |
| None (<25 battle deaths/year) | 0.00 | 0.00 |
| Minor conflict (25-999 battle deaths/year) | 13.14 (-22.59, 48.87) | - |
| War (≥1,000 battle deaths/year) | 78.27* (11.07, 145.5) | 51.18 (-6.78, 109.1) |
|  |  |  |
| **Covariates** |  |  |
| GDP per capita | 0.00 (-0.00, 0.00) | 0.00 (-0.00, 0.00) |
| OECD membership | -37.46 (-94.23, 19.32) | -40.42 (-98.77, 17.93) |
| Population density | -22.09 (-341.0, 296.8) | -19.46 (-345.2, 306.3) |
| Urbananisation | -16.51* (-30.93, -2.09) | -16.83* (-31.34, -2.32) |
| Age dependency ratio | -2.51 (-5.52, 0.50) | -2.68 (-5.69, 0.33) |
| Male education | -33.81 (-87.41, 19.80) | -35.85 (-89.31, 17.61) |
| Temperature | -7.06 (-24.81, 10.68) | -7.33 (-25.15, 10.49) |
| Rainfall | 22.72 (-9.77, 55.22) | 19.44 (-13.63, 52.51) |
| Earthquake | 5.52 (-15.78, 26.83) | 5.09 (-16.11, 26.29) |
| Drought | 1.01 (-14.25, 16.28) | 1.64 (-14.10, 17.38) |
| Observations | 4,754 | 4,754 |
| Countries | 183 | 183 |

**Note:** ^*^ *p* < 0.05, ^**^ *p* < 0.01, ^***^ *p* < 0.001. ^1^All-cause mortality without deaths from interpersonal violence; ^2^MEPV dataset replaces the armed conflict exposure with binary variable that captures the presence or absence of war. For more details on the MEPV dataset please see Additional file 1.

Robust standard errors were employed. Each column is the output from one panel regression with fixed effects adjusted for the covariates in the table in addition to year dummies (not shown). Coefficients are interpreted as the change in all-cause mortality per 100,000 following a change in one unit of the independent variable.

GDP per capita is in current US dollars. Population density represents the percentage of the population living in a density of >1,000 ppl/sqkm. Urbanisation represents the percentage of the population living in urban areas. The age dependency ratio represents the percentage of the population younger than 15 years and older than 64 years per 100 working-age population. Male education is expressed as years per capita and is age-standardised. Temperature is in degrees Celsius and is the mean population-weighted annual temperature. Rainfall represents the percentage of the population living in the top world quintile of annual rainfall. Earthquake and drought are binary variables representing their absence or presence. All armed conflict variables were lagged by one year.
